# Protection against Radiotherapy-Induced Toxicity

**DOI:** 10.3390/antiox5030022

**Published:** 2016-07-05

**Authors:** Susan Hall, Santosh Rudrawar, Matthew Zunk, Nijole Bernaitis, Devinder Arora, Catherine M McDermott, Shailendra Anoopkumar-Dukie

**Affiliations:** 1 Menzies Health Institute Queensland, Griffith University, Gold Coast 4222, Australia; s.hall@griffith.edu.au (S.H.); s.rudrawar@griffith.edu.au (S.R.); m.zunk@griffith.edu.au (M.Z.); n.bernaitis@griffith.edu.au (N.B.); d.arora@griffith.edu.au (D.A.); 2 School of Pharmacy, Griffith University, Gold Coast 4222, Australia; 3 Centre for Urology Research, Faculty of Health Sciences and Medicine, Bond University, Robina 4226, Australia; camcderm@bond.edu.au

**Keywords:** radiotherapy, toxicity, oxidative stress, inflammation

## Abstract

Radiation therapy is a highly utilized therapy in the treatment of malignancies with up to 60% of cancer patients receiving radiation therapy as a part of their treatment regimen. Radiation therapy does, however, cause a wide range of adverse effects that can be severe and cause permanent damage to the patient. In an attempt to minimize these effects, a small number of compounds have been identified and are in use clinically for the prevention and treatment of radiation associated toxicities. Furthermore, there are a number of emerging therapies being developed for use as agents that protect against radiation-induced toxicities. The aim of this review was to evaluate and summarise the evidence that exists for both the known radioprotectant agents and the agents that show promise as future radioprotectant agents.

## 1. Introduction

Radiation therapy is an important treatment modality for many malignancies with as many as 60% of cancer patients receive ionizing radiation as a part of their therapeutic regimen [1]. While radiotherapy has variable success depending on the cancer being treated, the toxicity or side effects associated with its use is of concern as it can affect quality of life [2]. Radiation toxicity can manifest in a number of different ways as summarized in Table 1 [3,4,5,6,7]. Adverse effects from radiation therapy are classified as early or late [3]. Early adverse effects occur during treatment or just after its completion, and usually resolve within four to six weeks [3]. Late adverse effects are observed several months to years after completion of treatment and may be permanent [3]. Another significant concern is the potential for secondary malignancies to occur, which usually manifest 10 to 15 years after treatment [8]. Therefore, there is considerable interest in agents that can serve as radioprotectants to potentially protect patients during treatment and prevent the substantial effects of toxicity.

### Direct and Indirect Mechanisms of Radiation-Induced Cell Damage

The nucleus is widely regarded as the principle target of ionizing radiation due to the fact that it is the largest organelle in the cell [9]. The biological effects of radiation can be direct and indirect. If energy from X- or γ-rays is absorbed in biological material, there is a possibility that it will interact directly with critical targets in the cell. The atoms of the target itself may be ionised or excited, leading to a chain of events culminating in biological damage. Alternatively, radiation may interact with molecules in the cell to produce reactive species, capable of damaging critical targets. This is referred to as an indirect action of radiation [9].

Ionizing radiation is an effective DNA-damaging agent, producing a range of lesions in cellular DNA, including more than 20 types of base damage, single-strand breaks, double-strand breaks, and DNA crosslinks [10]. Exposure of cells to clinical doses of ionizing radiation typically causes: 1000 single-strand breaks, 40 double-strand breaks and 3000 damaged bases per Gray (Gy) [11]. DNA double strand breaks are regarded as the most important for cell death [10]. Although cells have evolved complex repair mechanisms including homologous recombination and non-homologous end-joining, many breaks are not repaired or are misrepaired, generally leading to cell death [10]. As cells pass through the cell cycle, they have to progress through mitosis when cell division occurs, and cell death following irradiation occurs because of gross chromosomal changes leading to loss of genetic material from cells when they attempt to divide. In general, there is a close association between chromosomal fragments and cell killing by mitotic or reproductive death (failure to divide) [10]. However, cells respond to ionizing radiation-induce DNA damage by activating cell cycle checkpoints in order to allow for DNA repair, and prevent transmission of damaged DNA to daughter cells [10]. Furthermore, oxidation of protein and lipids induced by the free radicals produced by exposure radiotherapy is another major cause of healthy tissue damage [12,13].

In addition to direct ionisation of critical targets, ionizing radiation is able to induce cellular damage indirectly via the production of reactive species. This indirect mechanism accounts for approximately two thirds of the biological damage produced by X-rays, and is the central mechanism of the ”oxygen fixation hypotheses” [14]. The ”oxygen fixation hypothesis” is widely believed to account for the difference in radiation sensitivity between aerobic or normoxic and hypoxic cells [15]. Oxygen present at the time of irradiation, or within microseconds of the irradiation reacts chemically with the DNA radical produced by ionizing radiation and ”fixes” (makes permanent) the damage [16].

**Figure 1:**
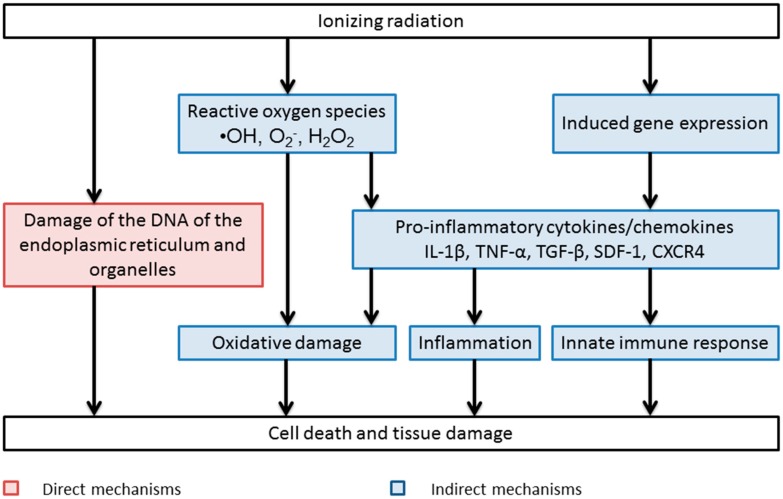
Direct and indirect mechanisms of cell death and damage associated with exposure to ionizing radiation.

In addition to free radical production, another mechanism of indirect toxicity post-irradiation is through an inflammatory process [17]. Post-irradiation, there is initiation of a pro-inflammatory reaction in the surrounding tissue resulting in the production of numerous pro-inflammatory cytokines and chemokines shortly after irradiation [17]. These include interleukin-1, interleukin-6, tumour necrosis factor α and transforming growth factor β [17]. TGF-β is of particular importance in pathophysiology of radiation toxicity particularly with regard to mediating radiation-induced fibrosis of the lungs and skin [18]. These mediators then initiate a long term inflammatory response and as result chronic inflammation and tissue injury occurs as seen in Figure 1 [17].

Low doses of irradiation are known to induce TGF-β resulting in potential extensive lung and skin damage [19]. This damage is mediated through changes to epithelial cell growth, a role of TGF-β [17]. Furthermore, TNF-α, primarily produced by activated macrophages, has been shown to play a crucial role in the acute phase of inflammation and has been shown to play a crucial role in radiation-induced fibrosis [17]. Interleukin-1, a key pro-inflammatory cytokine, works in conjunction with TNF-α to produce the skin reactions associated with radiation exposure [17]. Furthermore, given the pro-inflammatory nature of interleukin-1, it plays a critical role in mediating the inflammatory process post irradiation [17]. Inflammation post-radiation therapy is biphasic in response [20]. In the first two weeks post-radiation exposure, cytokine production is induced then returns to normal by the end of the two week mark [20]. The second increase in the cytokine expression occurs approximately six to eight weeks post-radiation therapy [20]. This indicates that the pro-inflammatory response is primarily responsible for the long-term toxicity associated with radiation toxicity, whereas the free radical production is associated with the short-term toxicities.

In addition to local effects, systemic effects occur after exposure to irradiation [21]. These effects include haematopoietic and immune cells resulting in decreases to the immune system [21]. Similar to local toxic effects of irradiation, systemic effects are mediated through oxidative stress, damage to DNA and the production of pro-inflammatory cytokines [22]. Phenotypic changes to macrophages occurs post irradiation, with cells changing from a predominantly anti-inflammatory state to a pro-inflammatory state thereby releasing pro-inflammatory cytokines and chemokines [22]. Furthermore, activation of NLRP3 inflammasome, a key mediator in the innate immune response and proceeding inflammatory response, has been shown to be critical in the systemic response post irradiation [22]. Other factors including the production of ATP, heat shock proteins and uric acid have also been shown to play a role [22]. The combination of some or all of these factors results in compromised cellular and tissue integrity ultimately leading to the deficits in immune system function and the haematopoietic cellular effects [22]. Methods for addressing this will be outlined in further detail below.

## 2. Measures to Minimise Radiation Damage

A number of measures exist to minimise tissue damage caused by radiation with many more emerging therapies having been identified. Current measures along with the major emerging therapies are outlined in Table 2 and will be discussed in detail below.

### 2.1. Clinically Used Radioprotectants

A number of clinically used radioprotectants have been identified and will be discussed below.

#### 2.1.1. Antioxidants

##### 2.1.1.1. Amifostine

Amifostine, a sulfhydryl-containing compound, has been shown to protect normal tissue during radiation therapy [23]. It is thought to do this via the scavenging of free radicals produced after exposure to radiation [23]. It is the most commonly used radioprotector clinically and is currently the only approved therapy used in radiation toxicity [24]. Amifostine has been evaluated for its effects against a number of pathologies of radiation inducing lung disease, mucositis, xerastomia and dysphagia [24]. 

To date, the most evidence lies with using amifostine preventatively for mucositis, xerastomia and dysphagia [24]. A recent meta-analysis showed that pre-treatment and treatment with amifostine both decreased the severity and the occurrence of all three of the manifestations associated with radiation therapy by up to 60% [24]. Other studies have shown positive effects on mucositis with the prophylactic use of amifostine [25], however, an earlier systematic review showed variability in the nature of results and design of the original studies, and as a result no recommendations could be made [26].

Another possible clinical use for amifostine in radiation-induced toxicity is for the associated lung damage. Lung damage commonly manifests as pulmonary fibrosis or pneumonitis after exposure to radiation therapy, especially in the upper regions of the body [82]. A number of in vivo studies have shown amifostine, when given before irradiation, protected the lungs against radiation toxicity [27,28]. In these studies, amifostine was dosed at 200 mg/kg and administered intraperitoneally [27,28].

Amifostine has been shown to have no effect on protecting the tumour cells against radiation. The accepted mechanism by which tumours remain sensitive to radiotherapy is due to the fact that amifostine is a pro-drug that requires alkaline phosphatase for conversion to its active form [83]. Tumour cells only express low levels of alkaline phosphatase and therefore only low levels of amifostine are converted to its active form [83].

##### 2.1.1.2. Glutamine

Glutamine, a non-essential amino acid, has been widely studied for its potential beneficial effects in a number of pathologies associated with radiation toxicity including mucositis, dermatitis and oesphagitis [29,30,31,32,33,34,35]. Glutamines proposed mechanism of action in the prevention of radiation toxicity is related to the formation of reduced glutathione (GSH) during its metabolism [84]. GSH is part of the antioxidant defences and is well known for its protective effects against free radical damage [84].

A recent meta-analysis investigated the role of glutamine therapy in the prevention of mucositis in radiation therapy [29]. It was shown that oral glutamine significantly decreased the incidence and duration of mucositis and other associated effects including weight loss [29]. Dosing regimens of the studies generally involved oral doses of 30 g/day that were administered in three divided doses [29].

Another common manifestation of radiation toxicity is dermatitis. Studies have shown glutamine to be beneficial in the prevention of radiation–induced dermatitis [30]. A recent study has shown that enteral glutamine not only reduced the severity of radiation-induced dermatitis but also significantly decreased the numbers of patients presenting with the radiation-induced dermatitis [30].

A small study (*n* = 32) evaluated the protective benefits of glutamine therapy in the prevention of oesphagitis associated with radiation therapy [31]. This study showed significantly less cases of oesphagitis in the patients receiving glutamine in comparison to control [31]. This study supports the findings of an earlier study showing that prophylactic dosing of glutamine at a dose of 10 g/8 h was beneficial in the prevention of oesphagitis [32].

Glutamine has however to date, not shown any benefit in preventing the occurrence of diarrhoea associated with radiation therapy [33]. This is further supported with a small randomized controlled trial examining the effects of glutamine on the occurrence diarrhoea associated with chronic enteritis [34]. This study showed no protective effect after glutamine was administered a dose of 30 g/day orally [34]. Another has shown glutamine to decrease the severity of diarrhoea associated with radiation therapy [35].

#### 2.1.2. Anti-Inflammatory Agents

##### Benzydamine

Benzydamine, a topical non-steroidal anti-inflammatory drug, has been evaluated for its use in preventing and treating oral mucositis associated with radiation therapy [40,41]. It is proposed to do this via its anti-inflammatory effects and more specifically through its inhibition of TNF-α [85]. A recent study has shown the effectiveness of benzydamine mouth rinse in preventing chronic oral mucositis [40]. This study showed that initiating benzydamine therapy one day prior to radiation therapy and continuing for two weeks post-exposure, had no effect on the incidence or severity of oral mucositis experienced by the patients at the Week 3 mark in comparison to placebo, however, this trend changed at the Week 4 and 7 marks post-radiation therapy [40]. At these time points benzydamine therapy decreased both the incidence and severity of oral mucositis in comparison to placebo [40]. This further supports previous studies that have shown benzydamine, applied topically, to be beneficial in decreasing the incidence and severity of oral mucositis associated with radiation therapy [41]. Again another small study (*n* = 14) has shown the benefit of using benzydamine mouthwash in oral mucositis after radiation therapy [42].

#### 2.1.3. Mixed Acting Agents

##### Pentoxifylline

Pentoxifylline, a methyl xanthine derivative, has been shown to possess immunomodulating, anti-inflammatory and vascular effects [36]. Pentoxifylline has been shown to be beneficial in decreasing the risk of radiation toxicity in the lung after oral administration [36,37,38,39].

A small number of studies have been undertaken assessing the effects of pentoxifylline on the pulmonary manifestations associated with radiation toxicity [36,37,38,39]. One study has shown pentoxifylline to have no effect on acute lung injury following radiation therapy but have shown it does however protect against chronic lung toxicity [37]. A number of other studies have, however, shown pentoxifylline to have beneficial protective effects against radiation toxicity in both acute and chronic toxicities [36,38]. These protective effects of pentoxifylline were observed when given orally at a dose of 400 mg three times a day [36].

Pentoxifylline is thought to exert its protective effects against radiation-induced toxicity due to its immunomodulating, anti-inflammatory and vascular effects [36]. In vivo studies suggest that pentoxifylline decreases TNF-α mRNA and protein production at a dose of 100 mg/day [39]. This may be the mechanism by which pentoxifylline exerts its protective effects given that TNF-α concentrations are increased at both a protein and transcriptional levels in the lungs [39]. Furthermore, prostaglandin E_2_, another marker of inflammation, is decreased in vivo when animals are pre-treated with pentoxifylline prior to radiation exposure [86]. In addition to its anti-inflammatory effects, pentoxifylline is proposed to protect against radiation-induced toxicities due to its antioxidant effects [86]. Studies have identified that pentoxifylline increases GSH and decreases lipid peroxidation in vivo post-radiation toxicity exposure [86]. This suggests that pentoxifylline has numerous mechanisms of action in the prevention of radiation-induced toxicities.

#### 2.1.4. Sulfasalazine

Sulfasalazine, a 5-aminosalicylate compound, has been evaluated for its effects in radiation therapy-induced enteritis. A number of studies found sulfasalazine to be beneficial in preventing the occurrence and reducing the severity of radiation-induced toxicity [43,44]. Sulfasalazine, administered as 1 g twice a day starting on the day of radiation therapy, was shown at the end of five weeks post-radiation exposure to significantly decrease acute gastrointestinal radiation toxicity as evidenced through endoscopic evaluation [44]. A larger study (*n* = 87) assessing the effects of sulfasalazine (1 g twice a day) found this therapy to be effective in decreasing the severity of diarrhoea associated with radiation therapy [43]. Sulfasalazine’s proposed mechanism of action in the prevention and severity reduction in radiation-induced toxicities is potentially two-fold. Not only is this an anti-inflammatory agent but is also a free radical scavenger [87].

### 2.2. Emerging Radioprotectors

As described earlier, there are only a limited number of agents that are used to prevent and manage the symptoms associated with radiation-induced toxicities, however, numerous agents have shown potential and will be outlined below.

#### 2.2.1. Natural Products as Radioprotective Agents

To date an abundance of natural products have been investigated as potential radioprotective agents. Endogenous compounds such as cytokines, hormones, amino acids, carbohydrates and various tissue extracts have been widely investigated [88,89,90]. Furthermore, numerous natural products have been studied for their effectiveness towards radiation toxicity and will be outlined below.

#### 2.2.2. Immunomodulators, Growth Factors and Cytokines

The emergence of recombinant technologies has enabled a large amount of research to be carried out in the area of engineered human immunomodulators ultimately leading to new therapeutic strategies. In the 1980s, it became apparent that endogenous compounds capable of non-specifically enhancing immunologic and haematopoietic responses could be utilised as radioprotectors [88,91]. The attention fell to cytokines and growth factors as new agents of radioprotection against radiation syndrome, and to date much work has been carried out to this end. Singh et al. noted studies have shown that although various cytokines have the ability to aid bone marrow restoration after treatment with cytotoxic drugs and exposure to radiation, not all possess the ability to be therapeutic after their complex interaction within the cytokine pathways they’re involved in are understood [88,89].

Presently, IL-1, TNF-α, SCF (mast cell growth factor), IL-12, the endogenous steroid family of 5-androstene and recombinant peptides derived from flagellin (CBLB502) and mycoplasma (CBLB601, CBLB6012 and CBLB6013) appear to significantly protect animals to varying degrees when administered prior to lethal doses of radiation [88,89]. A number of publications by Neta et al. studying the use of cytokines as radioprotectors has found the immunomodulator IL-1 produced a radioprotective effect when administered to mice prior (20 h) to whole body irradiation [91,92]. The protection was seen to be time and dose dependant in relation to irradiation, as noted, administration of IL-1 shortly after exposure to radiation did not have the same outcome with limited ability to save mice exposed from death. Furthermore, Neta et al. has hypothesised that the need for a lag phase between IL-1 administration and irradiation suggests that the radioprotection obtained from IL-1 is mediated by other agents induced by IL-1 therefore not by IL-1 itself directly [91]. This study presents evidence that conflicts the findings of the causes of radiation toxicity. As highlighted earlier, IL-1 and TNF-α have both been shown to play a crucial role in the inflammatory process post irradiation suggesting that further studies are required investigating these effects [17].

SCF has been shown to synergise in radioprotection with IL-1 as the release of IL-1 increases the amount of bone marrow cells expressing the endogenous receptor c-kit, which SCF is a natural ligand and thereby acts on [89]. However, the exact role that SCF plays in radioprotection is ambiguous currently, as numerous papers report conflicting experimental outcomes when SCF is used in combination with other cytokines and growth factors, which certainly further highlights the complex interplay between these endogenous compounds [89].

Several other cytokines and growth factors, including G-CSF, GM-CSF and erythropoietin (EPO), have been investigated as radioprotective agents. These agents act by stimulating the stem progenitor cells, promoting haematopoietic marrow repopulation [89,93]. However, these agents have been shown to be of limited use in radiotherapy [89], primarily due to pro-inflammatory and immunogenicity adverse effects, which in many cases (EPO) were seen to be severe [94].

Numerous other growth factors have been evaluated for their radiomodulating effects and furthermore drugs have been utilised that target these growth factors proving to have a further role in radioprotection. Anti-VEGF therapy using bevacizumab has been shown to decrease the extent of radiation necrosis in a small clinical trial of patients undergoing radiotherapy for brain tumours [95].

#### 2.2.3. Phyto-Radioprotectors

Phytochemicals produce their radioprotective effects through various mechanisms, with their activity being measured predominately as either antioxidants, free radical scavengers, DNA repair modulators or preventers of DNA damage and lastly based on anti-inflammatory action [96]. In the past 20 years, there has been a major shift towards evaluating phytochemicals as radioprotectors, primarily due to their potential bioequivalence, efficacy and in most cases low toxicity, relative to many of the established synthetic compounds available [97].

Plants are naturally instilled with radiation protective mechanisms as they rely on the harsh sunlight based radiation in order to grow. This innate radioprotective ability is in part due to the numerous antioxidant phytochemicals that they possess as part of normal metabolic processes. Polyphenols like flavonoids and their naturally occurring derivatives are structurally adapted in order to be activated by electron donating substituents which inhibit energy transfer mechanisms, ultimately suppressing oxidative stress and stabilising redox processing cells [98,99].

As can be seen in Table 2, antioxidant activity appears to be almost an essential characteristic of phytochemicals intended for radioprotection outcomes. Hence, the polyphenols make up the majority of compounds tested. These include all flavonoid based compounds, of which there are over 5000 recognised and their number is constantly increasing [90]. The use of polyphenols and the antioxidant lycopene are described in further detail below.

#### 2.2.4. Polyphenols as Radioprotectant Agents

Numerous polyphenols have been shown to have protective effects against radiation toxicities. Amongst these include curcumin, caffeic acid and ferulic acid which will be discussed in further detail below. These compounds have been shown to possess anti-inflammatory and antioxidant properties that are likely to target the indirect pathway of radiation toxicity [12,75,100,101,102,103,104,105,106,107,108].

Numerous studies have evaluated the radioprotective effects of curcumin both in vitro and in vivo. In vivo studies have shown curcumin to have protective effects against both acute and chronic skin toxicities when administered before or after exposure to radiation [100,101]. Furthermore, pre- or post-treatment with curcumin afforded a significantly decreased expression of pro-inflammatory cytokines and chemokines including IL-1, IL-6, TNF-α and TGF-β [100,101]. In vitro studies have shown similar results to in vivo studies. Curcumin has been shown to decrease lipid peroxidation and increase antioxidant effects in lymphocytes [102]. A limited number of human studies have evaluated the effectiveness of curcumin on reducing radiation-induced toxicity. One study has shown that oral administration of curcumin during radiotherapy afforded some protection against the severity of radiation-induced dermatitis in breast cancer patients [103]. A small randomised controlled trial (*n* = 40) showed curcumin to afford protection against urinary symptoms in patients receiving irradiation for prostate cancer [109]. It did not however provide any protection against bowel symptoms when dosed at 3 g/day [109]. Furthermore, a second randomised controlled trial found that 6 g/day of curcumin decreased radiation-induced dermatitis associated with breast cancer treatment [103].

Caffeic acid and caffeic acid phenethyl ester have shown promise in in vitro and in vivo studies assessing their protective effects in radiation-induced toxicity. In vivo studies have shown caffeic acid phenethyl ester to decrease lipid peroxidation and increase antioxidant defences in the heart and lung tissue of irradiated mice [104,105]. Furthermore, in vitro studies have shown caffeic acid to protect against radiation-induced DNA damage and lipid peroxidation in human peripheral blood lymphocytes [106].

Ferulic acid has been shown to possess anti-inflammatory and antioxidant activity indicating that its protective effects towards radiation toxicity are through targeting the indirect pathway of radiation toxicity [75]. In vitro studies have shown ferulic acid to protect against radiation toxicity predominantly via its antioxidant effects [12]. Pre-treatment of hepatocytes and lymphocytes with ferulic acid (1, 5 and 10 μg/mL) was shown to significantly decrease lipid peroxidation and damage to DNA after exposure to γ-irradiation [12,107]. Furthermore, it resulted in significant increases in antioxidant defences such as antioxidant enzymes and reduced glutathione (GSH) in comparison to the control group [12,107]. In vivo studies have also shown ferulic acid to be protective against γ-radiation toxicities [108]. Pre-treatment of mice with ferulic acid (50 mg/kg) 1 h prior to exposure to γ-radiation resulted in decreased DNA strand breaks in comparison to control [108].

The evidence provided suggests that polyphenols and their derivatives may provide significant improvement in radiation-induced toxicities, however, given the limited evidence in human randomized controlled trials, further studies are warranted before they are recommended for clinical use.

#### 2.2.5. Lycopene as a Radioprotectant Agent

Lycopene, a carotenoid, has been studied for its effects on a number of radiation-induced toxicity in both in vivo and in vitro models of radiation exposure. In vivo studies have shown lycopene to protect against radiation toxicity predominantly through an antioxidant pathway [110,111,112,113,114]. Treatment of rats with lycopene affords protection against increases in markers of lipid peroxidation and decreases in antioxidant defences in hepatic and small intestine tissue [110,111,112]. Similarly, lycopene was found to possess the same properties in an in vitro model of radiation toxicity in hepatic and lymphocytic cells [113,114].

#### 2.2.6. Other Emerging Radioprotectant Therapies

##### 2.2.6.1. Antioxidants

Endothelial cells (EC) are the most sensitive cells located in the vascular wall to radiation-induced ROS. Recent studies with human umbilical vein endothelial cells (HUVECs) demonstrate Vitamin D (Vit. D) protects endothelial cells from oxidative stress through modulating the mitogen-activated protein kinase (MAPK) signalling and the phospho-38 inactivation. Inactivation of p38 MAPK is an important mechanism by which potential radioprotective agents may exert their effects. Studies have shown that in oxygen-dependent irradiation, downstream activation of p38 MAPK is critical for damage to occur [115]. Furthermore, increases in oxidative stress results in mitochondrial permeability transition which leads to the activation of MAPK pathways post-irradiation [116]. This suggests a number of possible mechanisms by which Vit. D may exert its protective effects. Modulation of anti-apoptotic and anti-senescence MAPKs inhibits proteasome-mediated SirT1 protein degradation and resultant up-regulation of SirT1 protects EC from oxidant injury [52]. In addition, it has been observed that Vit. D deficiency is relatively common among patients with cancer [117] and phase III trials are currently evaluating radioprotective effect of Vit. D supplements [118]. It is important to note that many of the signal transduction pathways in the MAPK superfamily are involved in radiation-induced toxicity [119]. Furthermore, their role in inducing or protecting against radiation-induced toxicity is highly variable and is dependent on numerous factors including the cell type and the duration of exposure to radiation [119].

Melanin, a high-molecular weight natural pigment prevents formation of free radicals and scavenging of the highly destructive radicals generated by the radiolysis of water [120]. A recent study involving intravenous administration of melanin-coated nanospheres demonstrates protection of bone marrow against radiotoxicity during radioimmunotherapy of cancer [53]. Angiotensin I-Converting Enzyme Inhibitors (ACEIs) such as perindopril and captopril induces radioprotection by preserving murine haematopoietic short-term reconstituting cells in vivo [121]. Several mechanisms are involved in ACEIs’ haematopoiesis action. Primarily, ACEIs inhibit angiotensin II formation and increase plasma concentration of AcSDKP (acetyl-N-Ser-Asp-Lys-Pro), an ACE substrate and a negative regulator of haematopoiesis [122]. A recent clinical study demonstrated ACEIs significantly reduced the incidence, severity and duration of radiation-induced proctitis [54].

γ-Tocotrienol (GT3), a strong antioxidant [62], significantly protected haematopoietic tissue by preserving haematopoietic stem cells and progenitor cells and by preventing persistent DNA damage [123]. Further preclinical studies demonstrated GT3 conferred protection of intestinal cells from a GI-toxicity dose of radiation through upregulation of antiapoptotic and downregulation of proapoptotic factors, both at the transcriptional and the protein levels [60].

The modulation of the tetrahydrobiopterin pathway is gaining popularity in the potential treatment and prevention of radiation toxicity. Tetrahydrobiopterin is a cellular non-enzymatic redox sensitive antioxidant that is a co-factor in a number of biochemical pathways including aromatic amino acid hydroxylases and nitric oxide synthases [76]. Nitric oxide synthase, when exposed to increased conditions of oxidative stress, switches from producing nitric oxide to producing a number of free radicals including superoxide and peroxynitrite [77]. It is thought that inadequate supply of tetrahydrobiopterin is critical role in the switch to increased production of free radicals [77]. Furthermore, tetrahydrobiopterin has been shown to possess reactive oxygen species-scavenging activity suggesting that a deficit could contribute to radiation toxicity through a number of pathways [77,78]. Studies have shown that free radicals can decrease the concentrations of tetrahydrobiopterin [79]. This is thought to occur through dysregulation of the enzyme responsible for the homeostasis of tetrahydrobiopterin production, GTP cyclohydrolase I feedback regulatory protein (GFRP) [79,80,81]. This suggests that tetrahydrobiopterin or compounds that modulate the tetrahydrobiopterin pathway may be beneficial as a measure to prevent and/or treat radiation toxicity. 

##### 2.2.6.2 Emerging Anti-Inflammatory Agents

Entolimod, a toll-like receptor 5 (TLR5) ligand activates NF-κB signalling which inhibits apoptosis by suppression of p53 and upregulation of antiapoptotic cytokines [124]. This modulation of apoptosis is thought to play an important role in entolimod’s radioprotective activity [125]. In recent preclinical studies, entolimod demonstrated radioprotective activity in rodents and in non-human primates (rhesus macaques). A single dose intramuscular injection of entolimod enhanced morphological recovery of haematopoietic and immune system organs, decreased severity and duration of thrombocytopenia, anemia and neutropenia, and increased clonogenic potential of the bone marrow. Overall, entolimod reduced apoptosis and accelerated crypt regeneration in the gastrointestinal tract [58]. Entolimod is currently in clinical development as a medical radiation countermeasures (MRC) to reduce the lethality of high-dose total body ionizing irradiation.

Statins, or HMG-CoA reductase inhibitors, are another class of emerging therapies to treat radiation toxicities [67,68,69,70,71]. Again, statins predominantly exert their activities towards modulating the indirect pathway associated with radiation toxicity. In vivo studies have shown that lovastatin inhibited radiation-induced increases in pro-inflammatory mediators in lung tissue [67,68]. Furthermore, simvastatin and pravastatin administration was associated with protection of the gastrointestinal tract in vivo [69,70]. Similar results were observed in in vitro studies with pravastatin decreasing a number of pro-inflammatory cytokines including IL-6 and IL-8 in lung endothelial cells exposed to radiation [71].

##### 2.2.6.3. Mixed Acting Agents

Palifermin, an *N*-truncated recombinant human keratinocyte growth factor (KGF) protects the mucosal epithelium and promotes its early regeneration. Its protective actions appear to be due to multiple biological activities, which include inhibition of epithelial cell apoptosis and DNA damage, up-regulation of detoxifying enzymes and down-regulation of pro-inflammatory cytokines along with enhanced migration, proliferation and differentiation of epithelial cells [126]. Recent preliminary study confirmed clinical effectiveness of palifermin in prevention and treatment of oral mucositis in patients undergoing allogeneic stem-cell transplantation for the treatment of acute lymphoblastic leukaemia [55].

A superoxide dismutase transgene protects normal cells against radiation-induced cellular damage [127]. Manganese superoxide dismutase (MnSOD) transgene overexpression elevates antioxidant stores in tissues including levels of glutathione and decreases inflammatory cytokine production (IL-1, TNF-α and TGF-β) [128]. Preclinical investigation using intravenous manganese superoxide dismutase-plasmid liposomes (MnSOD-PL) administration demonstrated significant protective effect against ionizing radiation [56].

Genistein, a soy isoflavone protects cells against radiation-induced injury. The protective effect of genistein conferred through combination of cellular activities, including antioxidant activity, free radical scavenging activity and anti-inflammatory activity [129]. Preclinical studies demonstrated genistein protected haematopoietic progenitor cell recovery and survival [130]. In addition, recent clinical evidence indicates that genistein in conjugation with radiation therapy reduced the urinary, intestinal, and sexual adverse effects in patients with prostate cancer [57].

Another methyl xanthine derivative, caffeine, has been shown to have potential protective effects against radiation toxicity [72,73,74]. A cohort study in patients receiving radiation therapy for cervical cancer found an inverse correlation between the level of caffeine consumption at the time of the radiation therapy and the incidence of severe late radiation toxicity [73]. In vivo studies have evaluated the effectiveness of caffeine dosing on the survival of mice after exposure to lethal doses of γ-irradiation [72]. Caffeine afforded no protective benefits when dosed at the same time as irradiation; however, when dosed at high concentrations (80 mg/kg or 100 mg/kg) 30 or 60 min prior to irradiation, 50% and up to 70% survival at 25 days was observed, respectively [72]. Furthermore, caffeine was shown to decreased the severity of radiation-induced skin toxicities associated with radiation therapy [74]. Caffeine has been shown to possess anti-inflammatory and antioxidant properties [75]. These effects may contribute to the protective effects of caffeine in radiation-induced toxicity.

##### 2.2.6.4. Other Agents

PUMA, p53-Upregulated Mediator of Apoptosis plays an essential role in p53-dependent and DNA damage-induced apoptosis. On activation of PUMA by a wide range of apoptotic stimuli, PUMA directly binds to anti-apoptotic Bcl-2 like protein and initiates intrinsic apoptosis pathways [131]. Considering the importance of the interactions of PUMA/Bcl-2 like sproteins in initiating apoptosis, small molecules that disrupt or prevent these key interactions and consequently suppress PUMA induced apoptotic response have been investigated. In vitro analysis demonstrated several lead compounds conferred considerable protection against PUMA-dependent and radiation-induced apoptosis. Further work and optimization of the lead structures identified in this work required to develop PUMA inhibitors as safe and effective approach to protect radiation-induced tissue injury [59].

Recilisib, a chlorobenzyl sulfone derivative, is in clinical development as radiation countermeasure. Recent preclinical study indicates, recilisib administration after exposure to ionizing radiation enhances the recovery of haematopoietic cells by attenuating DNA damage response [60]. In addition, in vitro and in vivo studies on mouse and human bone marrow cell demonstrated recilisib effectively protects cells from radiation-induced damage. Further mechanistic studies showed recilisib confers its protective effect through the up-regulation of Pl3-Kinase/AKT pathways in cells exposed to radiation [61].

In recent preclinical study, apart from a potent antioxidant activity, the Vitamin E isoform δ-Tocotrienol (DT3) demonstrated radioprotection by enhancing haematopoiesis and modulating signalling pathways. Further investigation of radioprotection mechanism indicates DT3 reduced activation of caspases (caspases 3, 7 and 8) inherent to apoptosis, while increasing autophagy-related to beclin-1 expression in irradiated bone marrow [65]. DT3 significantly increased haematopoietic progenitor cell survival and regeneration of haematopoietic microfoci and progenitor cells in irradiated mouse bone marrow. In addition, DT3 also protected human CD34^+^ cells from radiation-induced damage through activation of extracellular signal-related kinase 1/2 (Erk1/2) phosphorylation and significant inhibition of formation of DNA-damage marker γH2AX foci [64].

Recent evidence indicates that DIM (3,3′-Diindolylmethane) protected cultured cells against ionizing radiation by a unique mechanism: DMI activated an Ataxia telangiectasia mutated (ATM)-driven DNA damage (DDR)-like response without causing DNA damage and induced NF-kB signalling [132].

Collectively, these novel approaches will hopefully lead to development of safe and effective radioprotective agent [83,133,134].

## 3. Conclusions

In conclusion, radiation toxicity is a major problem for patients receiving therapy for malignancies. To date, there are only a limited number of radioprotectant agents used clinically to minimise the severity and duration of toxicities associated with radiation therapy. There are a number of promising agents emerging, however, further studies assessing their effects is required. This review has highlighted that there is a lack of high quality human studies for many of the agents described above. In particular, further studies in humans are required to evaluate the safety and efficacy of these emerging therapies in not only the prevention and treatment of radiation toxicity but also their effects on tumour sensitivity.

## Figures and Tables

**Table 1:** List of adverse effects associated with radiation therapy and the associated cancers [3].

Adverse Effect	Associated Cancer	References
Depression	Breast, lung, pancreatic, oropharyngeal, brain	[3,4,5]
Fatigue	Brain, head and neck, breast, lung, pelvic lymphatic system	[3,5]
Dermatitis	Head and neck, breast, prostate, perineal	[3]
Cardiovascular disease	Hodgkin lymphoma, breast, lung	[3,6]
Pneumonitis	Breast, lung, mediastinal	[3]
Xerostomia	Head and neck	[3]
Mucositis and esophagitis	Head and neck, thoracic	[3]
Enteritis	Abdominal, pelvic	[3]
Proctitis	Anal, rectal, cervical, uterine, prostate, bladder, testicular	[3]
Emesis	Upper abdominal, craniospinal, pelvic	[3]
Cystitis	Prostate, colorectal, bladder, pelvic	[3,7]
Erectile dysfunction	Prostate, colorectal	[3]
Vaginal dryness and stenosis	Cervical, endometrial, vaginal	[3]
Infertility and teratogenicity	Cervical, pelvic, testicular	[3]

**Table 2:** A summary of radioprotectant compounds.

Compound	Mechanism of Action	References
*Clinically used radioprotectants*		
Amifostine	Antioxidant	[23,24,25,26,27,28]
Glutamine	Antioxidant	[29,30,31,32,33,34,35]
Pentoxifylline	Antioxidant, anti-inflammatory	[36,37,38,39]
Benzydamine	Anti-inflammatory	[40,41,42]
Sulfasalazine	Anti-inflammatory, antioxidant	[43,44]
*Emerging Radioprotectants—Natural Products*		
Curcumin (*Curcuma longa*)	Antioxidant, anti-inflammatory, antiproliferatiive	[45]
Quinic acid (Coffee, cocoa)	Antioxidant, decreases DNA damage	[46]
lycopene (*Lycopersicon esculentum*)	Antioxidant, peroxidation inhibitor, free radical scavenger	[47]
Rutin (bioflavonoid)	Antioxidant	[48]
Hemocyanin (*Rapana thomasiana*)	Radiomitigator	[49]
Black tea extract *(Camellia sinensis)*	Free radical scavenger	[50]
Silymarin *(Silybum marianum)*	Anti-apoptotic agent, Reduces DNA damage	[51]
*Other Emerging Therapies*		
Vitamin D	Protection of endothelial cells by modulating MAPK/SirT1 axis	[52]
Melanin nanoparticles	Protection of haematopoietic cells by preventing generation of free radicals and of free radical scavenging	[53]
Angiotensin-I-Converting Enzyme (ACE) inhibitors -Captopril	Haematopoietic radioprotection mediated by angiotensin II pathway through AT1 receptors	[54]
Palifermin	Stimulation of cell proliferation and inhibition of cell apoptosis	[55]
Manganese superoxide dismutase-plasmid liposome (MnSOD-PL) gene therapy	Antioxidant, decreases free radical production and inflammatory cytokine release	[56]
Genistein	Antioxidant and anti-inflammatory	[57]
Entolimod	Apoptosis modulation	[58]
PUMA inhibitors	Apoptosis modulation	[59]
Recilisib	Intracellular signalling and DNA damage repair pathways P53 down-regulation and up-regulation of AKT pathways	[60,61]
γ-Tocotrienol	Antioxidant, free-radical scavenger, HMG-CoA reductase inhibitor	[62,63]
δ-Tocotrienol	Antioxidant, enhance haematopoiesis, modulate signalling pathways	[64,65]
3,3′-Diindolylmethane	Induces ATM-dependent DDR-like response, enhances radiation-induced ATM signalling and NF-κB activation	[66]
Statins	Anti-inflammatory	[67,68,69,70,71]
Caffeine	Antioxidant and anti-inflammatory	[72,73,74,75]
Tetrahydrobiopterin	Modulation of free radical-induced damage	[76,77,78,79,80,81]
